# A Scoping Review on Exercise Interventions During Adjuvant Chemotherapy or Radiotherapy for Cancer

**DOI:** 10.7759/cureus.106295

**Published:** 2026-04-01

**Authors:** Tushara Nair, Chakshu Mehta, Palani G Kumar

**Affiliations:** 1 College of Physiotherapy, Sumandeep Vidyapeeth Deemed to be University, Vadodara, IND

**Keywords:** adjuvant therapy, cancer, chemotherapy, exercise, physiotherapy, radiotherapy, rehabilitation

## Abstract

Cancer is one of the most common causes of death worldwide. Surgery, chemotherapy, and radiotherapy remain the first line of treatment for many different types of cancer. The diagnosis of cancer and its treatment lead to many physical and psychological symptoms. Several types of exercise interventions are available to help alleviate these symptoms. However, there is very little evidence available that provides an overview of all the exercises and outcomes. Hence, the aim of this review is to provide an overview of the various exercise interventions and their outcomes for patients with cancer undergoing adjuvant therapy. PubMed and Physiotherapy Evidence Database (PEDro) were searched with the following Medical Subject Headings (MeSH) terms: “Physiotherapy” OR “Physical therapy” OR “Exercise” AND “Adjuvant therapy for cancer” for the last 10 years. Full-text articles describing exercise interventions for patients receiving adjuvant therapy for cancer were included. A total of 40 articles were included in the analysis. The findings of this review suggest that aerobics, strengthening exercises, and the combination of both are the most common types of interventions used. A multimodal therapy that includes aerobics, strengthening, balance, mobility, and flexibility exercises is also commonly used. Apart from this, yoga and relaxation are also being used in cancer patients. The frequently used outcome measures include fatigue, cardiorespiratory fitness, and quality of life (QoL), followed by physical fitness, anthropometric measurements, anxiety and depression, cognitive function, and others. Thus, this review provides an overview of current exercise trends and outcome measures in practice for patients with cancer undergoing adjuvant therapy.

## Introduction and background

Cancer is defined as a condition in which the normal cells of the body divide uncontrollably and infiltrate the normal tissues of the body [1]. According to Global Cancer Statistics 2020, the overall disease load is increasing globally as well as in India, with 19.3 million incident cancer cases worldwide in 2020 [2]. These statistics are alarming and indicate a need to improve cancer care.

Surgery and adjuvant therapy in the form of chemotherapy, radiation therapy, and chemoradiation therapy form the major line of treatment. The pathophysiology of the disease and the treatment options lead to many physical and psychological symptoms, such as cancer-related fatigue (CRF), reduced cardiorespiratory and physical fitness, reduced cognitive levels, cancer-specific complications, depression, and anxiety, which reduce the quality of life (QoL) [3].

Exercise creates a sense of well-being, which helps in reducing the above symptoms [4]. Exercise interventions and outcome measures for patients with cancer vary widely across different stages of treatment. Numerous systematic reviews have examined specific types of exercise interventions and their effects within the cancer population. However, there remains a lack of comprehensive overviews focusing on the range of exercise interventions used specifically during ongoing adjuvant therapy. Therefore, the aim of this study was to identify and provide a comprehensive overview of the exercise interventions and outcome measures that are currently commonly used in patients undergoing adjuvant cancer therapy.

## Review

Methods

The objectives of the study were to identify different types of exercises and outcome measures available for patients receiving adjuvant therapy for cancer, and to thoroughly explore this broader aspect, a scoping review approach was used. This review followed a standardized and meticulous protocol given by Arksey and O’Malley [5]. The "Preferred Reporting Items for Systematic Reviews and Meta‐analyses (PRISMA) extension for Scoping Reviews” checklist was used to report the findings of this study [6].

Research questions 

The research question was formulated after an extensive search of the literature and identifying the dearth in the same. The PCC (Population/Concept/Context) framework was used to define the research questions proposed by the Joanna Briggs Institute [7]. The following were the research questions: (i) What is the scope and variety of articles published on cancer rehabilitation? (ii) What types of exercise interventions are currently used in patients receiving adjuvant therapy for cancer? (iii) What outcome measures are used to assess the effects of exercise interventions in patients undergoing adjuvant therapy? (iv) What exercise interventions are specifically implemented during ongoing adjuvant therapy? (chemotherapy + radiotherapy + chemoradiation therapy). Based upon these research questions, the following hypotheses were made: (i) There is a broad and diverse range of published literature on cancer rehabilitation, reflecting increasing research interest in this field. (ii) Multiple types of exercise interventions (e.g., aerobic, resistance, and combined training) are currently used in patients receiving adjuvant therapy. (iii) A variety of outcome measures, including physical, functional, and quality-of-life indicators, are used to evaluate the effects of exercise interventions. (iv) Structured and tailored exercise interventions are being specifically implemented during ongoing adjuvant therapy, though their application may vary across studies. Initially, Indian context articles were supposed to be included, but there was very little to no literature available. The research question was furthermore broadened.

Search strategies

The online databases PubMed and PEDro were searched with the following MeSH terms and Boolean operators: “Physiotherapy” OR “Physical therapy” OR “Exercise” AND “Adjuvant therapy for cancer” for the last 10 years from 2015 to 2025. The articles were first scrutinized based on the titles and abstracts, then the full-text articles were analyzed based on the inclusion and exclusion criteria.

Selecting relevant studies

**Figure 1:**
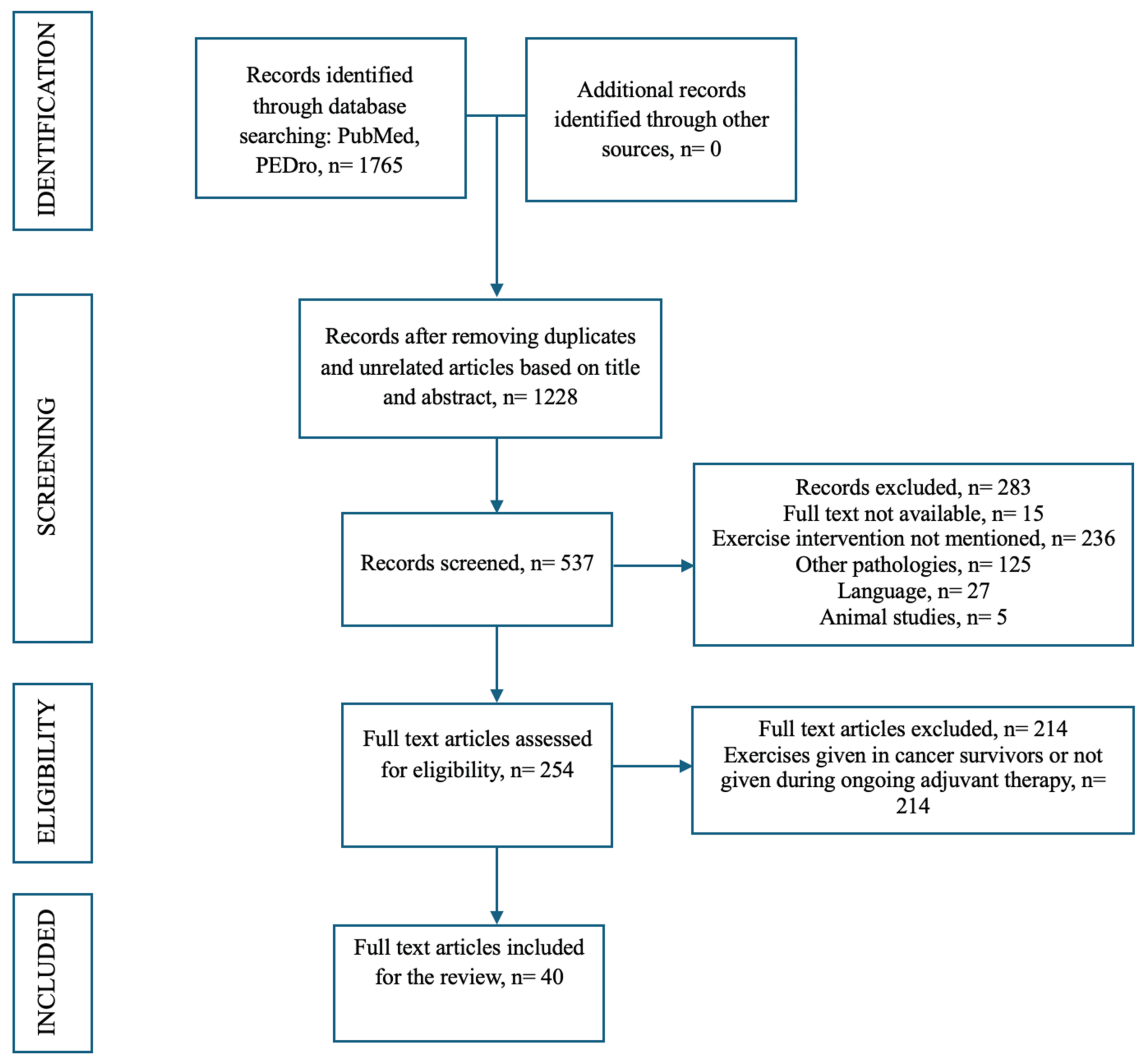
PRISMA flow diagram.

All the full-text articles focusing on physiotherapy or any exercise intervention in cancer patients receiving adjuvant therapy, which is chemotherapy, radiation therapy, or chemoradiation therapy after surgical treatment, written in English, were included. Randomized controlled trials (RCTs), non-randomized trials, quasi-experimental, cross-sectional, observational, case studies, and case series were considered. Articles where exercise intervention was not mentioned, or exercises given after completion of adjuvant therapy, or exercises given to cancer survivors or patients receiving neoadjuvant chemotherapy were excluded. Ongoing trials, animal experiments, conference abstracts, reviews, editorials, and study protocols were also excluded. After a detailed evaluation of the articles, 40 articles were included for this scoping review. Two authors independently extracted the data, and the disagreements were solved after discussion with the third author. The search results are described in the flow diagram in Figure 1.

Data extraction

After the inclusion, to identify the exercise interventions and outcomes, a data extraction sheet was developed that included the following details: study author, year, title of study, type of study, study population, intervention, outcome measures used, and results. The intensity and duration of exercises were not taken into consideration, as the primary objective was to find out the different types of exercise interventions available for patients undergoing adjuvant therapy for cancer. The instruments utilized for the outcomes were again not included, as the main aim was to identify the common outcomes that are affected by the exercises. The first author extracted all the data, which was verified by the co-authors.

Critical appraisal

According to the guidelines for administering scoping reviews, critical appraisal of the articles for errors in methodology is not necessary [7]. Hence, all the selected articles were included. 

Results

PubMed and PEDro were thoroughly searched, with the MeSH terms provided above, which rendered 1765 articles. Then the duplicate articles and the unrelated articles based on title and abstract were removed, which left 1228 articles. After this, the records were further screened for the eligibility criteria, and a total of 40 articles were included in the study. The data extracted from these articles are represented in Table 1.

**Table 1:** Summary of articles included.

Author (Year)	Title	Study design	Participants	Intervention	Outcome measures	Results and Conclusion
Moreira T R et al. [8] (2025)	Effects of an Easily Implemented Physical Exercise Program on the Ventricular Ejection Fraction of Women with Breast Cancer Undergoing Chemotherapy	Non-randomized control trial	38 breast cancer patients undergoing chemotherapy	Supervised physical exercise program (Joint mobilization, aerobic training, strength training)- Experimental training, Advice to remain active- Control group	Left ventricular ejection fraction (LVEF)	Exercise group- slight reduction in LVEF. Control group- Significant reduction in LVEF suggestive of protective effects of exercise.
Nair T et al. [9] (2025)	Effect of an Eclectic Physiotherapeutic Approach on Physical Performance, Fatigue, and Hospital Anxiety and Depression in a Patient Receiving Radiotherapy for Squamous Cell Carcinoma of the Buccal Mucosa: A Case Report	Case report	1 patient with squamous cell carcinoma of buccal mucosa receiving radiotherapy	Personalized protocol which included General mobility, strengthening, light aerobics, relaxation and recreational activities	Physical performance, fatigue, hospital based anxiety and depression	Positive improvements in all the outcome measures.
Moser R et al. [10] (2024)	A survey of cancer patients’ interest in undertaking exercise to promote relaxation during radiotherapy for breast cancer and metastatic cancer	Prospective pseudonymized survey	100 patients receiving radiotherapy for breast cancer	Relaxation exercises/ interventions	Self-developed 11 item questionnaire	Patients desire relaxation exercises to relieve stress and anxiety from radiotherapy.
Strijker D et al. [11] (2024)	Multimodal rehabilitation (Fit4Chemo) before and during adjuvant chemotherapy in patients with colonic cancer	Prospective interventional study	16 stage 3 colon cancer patients	Multimodal rehabilitation program (Fit4Chemo)	Primary outcomes- Feasibility, safety, practicality, Secondary outcomes- Physical fitness, physical function, and body composition	Feasibility was good. Secondary outcomes improved in 1st phase and remained unchanged in 2nd phase. The exercise intervention was safe, feasible and was found to improve cancer outcomes.
Anandavadivelan P et al. [12] (2024)	Five‐year follow‐up of the OptiTrain trial on concurrent resistance and high‐intensity interval training during chemotherapy for patients with breast cancer	RCT	179 breast cancer survivors who had received exercises during chemotherapy	Concurrent resistance and high-intensity interval training (RT-HIIT) or concurrent moderate-intensity aerobic exercise and high- intensity interval training (AT-HIIT)- Experimental group, Usual care- Control group	Primary outcome: CRF, Secondary outcomes were symptoms and symptom burden, HRQOL, cardiorespiratory fitness, lower and upper body muscle strength, pressure pain threshold (PPT), objective measures of sedentary behaviour and physical activity, body mass, sick leave, heart failure diagnosis and cognitive functioning.	The effect of RT-HIIT for the primary and secondary outcomes are limited in the long-term.
Inbaraj G et al. [13] (2023)	Effects of an 18-Week Integrated Yoga Program on Cardiac Autonomic Function in Breast Cancer Patients Undergoing Adjuvant Chemotherapy: A Randomized Controlled Trial	RCT	68 breast cancer patients receiving adjuvant chemotherapy	Yoga therapy- Experimental group, Usual treatment- Control group	Resting heart rate (RHR), Heart Rate Variability (HRV)	Higher RHR and lower HRV indices in control group which showed protective effects of Yoga therapy in CAD patients.
Brown M et al. [14] (2023)	Feasibility of delivering supervised exercise training following surgical resection and during adjuvant chemotherapy for pancreatic ductal adenocarcinoma (PRECISE): a case series	Case series	8 patients with pancreatic ductal carcinoma receiving adjuvant chemotherapy	Personalized, supervised, progressive exercise program	Feasibility, Anthropometric outcomes, Physical fitness outcomes, Patient-reported outcomes	Exercises were found to be feasible and safe and prevented an expected decline in the functional fitness and the patient reported outcomes.
Moulton C et al. [15] (2023)	Online Home-Based Physical Activity Counteracts Changes of Redox-Status Biomarkers and Fitness Profiles during Treatment Programs in Post-surgery Female Breast Cancer Patients	RCT	20 patients with breast cancer scheduled for adjuvant therapy	Online exercise training program (aerobics + resistance exercises)- Experimental group, Usual care- Control group	Blood Sampling and Isolation of Peripheral Blood Mononuclear Cells (PBMCs)	Exercise intervention improves functional and anthropometric parameter (free fat mass and fat mass) and activates cellular responses.
Naaktgeboren WR et al. [16] (2023)	Effects of physical exercise during adjuvant chemotherapy for breast cancer on long‐term tested and perceived cognition: results of a pragmatic follow‐up study	Pragmatic follow-up study	143 breast cancer patients who had received chemotherapy	Supervised, low intensity exercises, moderate to high intensity exercise- Experimental group, Usual care (medical and nursing care)- Control group	Cognitive outcomes- Online Amsterdam Cognitive Scan (ACS)	No significance between groups
Greaney SK et al. [17] (2022)	Yoga Therapy During Chemotherapy for Early-Stage and Locally Advanced Breast Cancer	RCT	30 breast cancer patients undergoing adjuvant therapy	Yoga therapy- Experimental group and Routine breast cancer treatment- Control group	Feasibility, Weight change, mood, fatigue, QOL, TNF-alpha, CRP	Decrease in body weight in the experimental group, no change in the other outcome measures. Yoga was beneficial to improve QOL and in weight maintenance.
Liu W et al. [18] (2022)	Effect of mindfulness yoga on anxiety and depression in early breast cancer patients received adjuvant chemotherapy: a randomized clinical trial	RCT	136 breast cancer patients receiving adjuvant chemotherapy	Mindfulness yoga+ conventional care- Experimental group, Conventional care- Control group	Hospital anxiety and depression, fatigue, pain, health related quality of life	Significant difference was found in anxiety, depression and QOL, no difference fatigue and pain
Van der Schoot G G F et al. [19] (2022)	Optimal Timing of a Physical Exercise Intervention to Improve Cardiorespiratory Fitness During or After Chemotherapy	RCT	266 patients with cancer receiving chemotherapy	Group A- Supervised exercise during chemotherapy, Group B- Supervised exercise program after chemotherapy	VO2 peak postintervention, muscle strength, health-related quality of life (HRQoL), fatigue, physical activity, and self-efficacy	Exercises can be performed safely during chemotherapy and it shows less decreases in VO2 peak , HRQoL, and muscle strength and reported less fatigue and more physical activity in intervention group than control group.
Lund C M et al. [20] (2021)	The effect of geriatric intervention in frail older patients receiving chemotherapy for colorectal cancer: a randomized trial (GERICO)	RCT	142 colorectal cancer patients receiving chemotherapy	Comprehensive geriatric assessment (CGA) based intervention- Experimental group, Standard care- Control group	Completion of planned chemotherapy course, toxicity, survival, QOL	Experimental group patients were able to complete the chemotherapy dose better compared to control group. Exercises improve the burden of illness and mobility.
Zopf E M et al. [21] (2021)	Effects of supervised aerobic exercise on cardiorespiratory fitness and patient‐reported health outcomes in colorectal cancer patients undergoing adjuvant chemotherapy—a pilot study	Pilot study	59 colorectal cancer patients receiving adjuvant chemotherapy	Supervised aerobic exercise program- Experimental group, Usual care- Control group	Cardiorespiratory fitness, fatigue, QOL, physical activity levels	Significant improvements in cardiorespiratory fitness and mental fatigue.
Vincent F et al. [21] (2020)	Home-Based Physical Activity in Patients With Breast Cancer: During and/or After Chemotherapy? Impact on Cardiorespiratory Fitness. A 3-Arm Randomized Controlled Trial (APAC)	RCT	81 breast cancer patients receiving chemotherapy	Group A: Home-based Adapted Physical Activity (APA) program during adjuvant or neoadjuvant therapy Group B: Home-based APA program after adjuvant or neoadjuvant therapy Group C: Home-based APA program during and after adjuvant or neoadjuvant therapy	Cardiopulmonary exercise tests, Six-minute walking test , Body composition, Peripheral muscular strength Fatigue, QOL, Anxiety and depression, Physical activity level	VO2 peak increased during the exercise duration and decreased during the chemotherapy period without APA also positive effects on CRF and physical functions were found.
Henriksson A et al. [22] (2020)	Is it safe to exercise during oncological treatment? A study of adverse events during endurance and resistance training – data from the Phys-Can study	Descriptive and comparative	577 patients participated in exercises, 90 patients in usual care (breast cancer patient planned to receive neo/adjuvant chemotherapy)	High intensity or low moderate intensity exercises	Adverse events (AE)	20% of participants had AEs, 28% had self-reported AEs, High intensity had higher likelihood of AEs.
Toth M J et al. [23] (2020)	Effect of neuromuscular electrical stimulation on skeletal muscle size and function in patients with breast cancer receiving chemotherapy	RCT	17 breast cancer patients scheduled for chemotherapy	Neuromuscular Electrical Stimulation (NMES)- Experimental group, Usual care- Control group	Muscle tissue processing, Immunohistochemistry, Skeletal muscle fiber contractile function, Body weight and composition, 6-Min walk test, Knee extensor muscle function, Physical activity.	Muscle fibre hypertrophy, muscle fibre shifts seen with minimal effects in muscle fibre contractility and reductions in sarcolemmal mitochondria.
Hiensch AE et al. [24] (2020)	Inflammation Mediates Exercise Effects on Fatigue in Patients with Breast Cancer	RCT	240 breast cancer patients undergoing chemotherapy	Resistance and high-intensity interval training (RT-HIIT)- Group A, Moderate-intensity aerobic and high-intensity interval training (AT-HIIT)- Group B, or usual care- Control group	Fatigue, 62 markers (IL-6 TNF- alpha)	Significant decrease in fatigue and significantly reduced inflammation in exercise group
Moller T et al. [25] (2020)	Physical deterioration and adaptive recovery in physically inactive breast cancer patients during adjuvant chemotherapy: a randomized controlled trial w	RCT	153 breast cancer patients receiving chemotherapy	Supervised hospital-based group exercise program- Group 1, Home-based individual pedometer- Group 2, Physically inactive patients- Control group.	Primary outcomes- CRF, VO2 peak	Cardiorespiratory fitness improved in both the groups, but there was no significant differences in between two groups.
Febvey-Combes O et al. [26] (2020)	Effects of an Exercise and Nutritional Intervention on Circulating Biomarkers and Metabolomic Profiling During Adjuvant Treatment for Localized Breast Cancer: Results From the PASAPAS Feasibility Randomized Controlled Trial	RCT	61 breast cancer patients receiving adjuvant chemotherapy	Aerobic exercises with nutritional counselling- Experimental group, Usual care- Control group	Biomarkers (insulin, insulin-like growth factor 1, estradiol, adiponectin, leptin, interleukin-6, and tumor necrosis factor α)	No statistically significant difference was found.
Simonsen C et al. [27] (2020)	Effects of high-intensity exercise training on physical fitness, quality of life and treatment outcomes after esophagectomy for cancer of the gastro-esophageal junction: PRESET pilot study	Pilot study	49 gastro-esophageal junction cancer patients receiving adjuvant or neoadjuvant chemotherapy	Supervised high-intensity aerobic and endurance training- Experimental group, Usual care- Control group	Cardiorespiratory fitness, muscle strength, body composition, HRQOL, body weight	Improvements in muscle strength and cardiorespiratory fitness. At 1 year follow up, no differences in HRQOL was found in between the groups.
Jacot W et al. [28] (2020)	Brief Hospital Supervision of Exercise and Diet During Adjuvant Breast Cancer Therapy Is Not Enough to Relieve Fatigue: A Multicenter Randomized Controlled Trial	RCT	360 breast cancer patients receiving adjuvant radiotherapy and chemotherapy	Adapted Physical Activity Diet (APAD) education program- Experimental group, Usual care- Control group	Fatigue	No significant difference between the groups
Feyzioglu O et al. [29] (2019)	Is Xbox 360 Kinect-based virtual reality training as effective as standard physiotherapy in patients undergoing breast cancer surgery?	RCT	40 breast cancer patients receiving adjuvant chemotherapy	Kinetic based rehabilitation group (KBRG) (Group A)- Xbox Kinetic-based games, Standardized physical therapy group (SPTG)- Group B	Pain, grip strength, functionality, muscle strength, ROM, and fear of movement	Significant improvements in functionality in KBRG
Troschel F M et al. [30] (2019)	High-Intensity Physical Exercise in a Glioblastoma Patient under Multimodal Treatment	Case-study	1 patient with Glioblastoma	High-Intensity physical exercise	Exercise duration	Exercises are feasible even in long-term without adverse events and gain in fitness was seen.
Hiraoui M et al. [31] (2019)	Effects of combined supervised intermittent aerobic, muscle strength and home-based walking training programs on cardiorespiratory responses in women with breast cancer	RCT	32 breast cancer patients undergoing chemotherapy	Supervised intermittent cycling aerobic, muscle strength and home-based walking training programs- Experimental group, Usual care- Control group	Walking speed (WS), walking distance covered (WD), heart rate (rHR), blood lactate ([La]b) concentration and rating of perceived exertion (RPE)	Significant increase in all the outcomes in the exercise group.
Carayol M et al. [32] (2019)	Short- and long-term impact of adapted physical activity and diet counseling during adjuvant breast cancer therapy: the “APAD1” randomized controlled trial	RCT	143 breast cancer patients undergoing chemotherapy or radiotherapy	Moderate intensity mixed aerobic and resistance exercises and diet consultations- Experimental group, Usual care- Control group	Fatigue, QOL, Anxiety and Depression	Significant improvements in all the outcomes.
Hatlevoll I et al. [33] (2019)	Physical exercise during adjuvant chemotherapy for colorectal cancer—a non-randomized feasibility study	Non-randomized interventional feasibility study	19 colorectal cancer patients undergoing chemotherapy	Tailored exercise program (progressive aerobic endurance, resistance and balance exercises)	Feasibility, Chemotherapy induced peripheral neuropathy (CIPN), fatigue	Feasibility was good, with no major adverse events, improvements in patient reported symptoms of CIPN and fatigue
Maurer T et al. [34] (2019)	Randomized controlled trial testing the feasibility of an exercise and nutrition intervention for patients with ovarian cancer during and after first-line chemotherapy (BENITA-study)	RCT	32 ovarian cancer patients receiving adjuvant chemotherapy	Personalized exercises (endurance, aerobic and balance exercises) and nutritional counselling- Experimental group, Usual care- Control group	Recruitment rate, adherence, completion and adverse events to the protocol, HRQOL, CRF, muscle quality and function, dietary intake and quality	Feasibility of the study was good, based on the safety and acceptance of exercise and nutrition intervention. Large multicentric RCT is in plan to investigate the effects on other outcomes.
Foucaut A et al. [35] (2019)	Feasibility of an exercise and nutritional intervention for weight management during adjuvant treatment for localized breast cancer: the PASAPAS randomized controlled trial	RCT	61 breast cancer patients receiving adjuvant chemotherapy	Aerobic exercises with nutritional counselling- Experimental group, Usual care- Control group	Adherence	Good adherence as the patients completed the intervention duration.
Kirkham A A et al. [36] (2018)	Effectiveness of Oncologist-Referred Exercise and Healthy Eating Programming as a Part of Supportive Adjuvant Care for Early Breast Cancer	Prospective, single-arm intervention program	73 breast cancer patients receiving adjuvant therapy	NExT program (Nutrition and exercise during Adjuvant Treatment)- Supervised moderate intensity, aerobic and resistance exercises	Moderate-to-vigorous physical activity (MVPA) and HRQoL	During treatment, increase in MVPA but no change in HRQoL. After one year, increase in both outcomes.
Witlox L et al. [37] (2018)	Four-year effects of exercise on fatigue and physical activity in patients with cancer	RCT	128 breast and colon cancer patients who had received exercises during adjuvant therapy	Supervised aerobic and muscle strength exercise program- Experimental group, Usual care- Control group	Fatigue, physical activity	Significant positive effects on fatigue and physical activity immediate post intervention, but at 4 years post intervention only fatigue showed significant intervention in the intervention group.
Schmidt T et al. [38] (2017)	Influence of physical activity on the immune system in breast cancer patients during chemotherapy	RCT	81 patients with breast cancer receiving chemotherapy	Supervised resistance training or endurance training- Experimental group, Usual care- Control group	CD3+ T lymphocytes	Chemotherapy lead to decrease in the immunity. Exercises did not further suppress the immunity as there was no significant difference in between the groups.
Mostarda C et al. [39] (2017)	Short-term combined exercise training improves cardiorespiratory fitness and autonomic modulation in cancer patients receiving adjuvant therapy	RCT	18 breast cancer patients receiving adjuvant therapy	Short-term combined exercise training (Resistance+aerobic+flexibility)- Experimental group, Usual care- Control group	Cardiorespiratory fitness and autonomic modulation	Reverses cardiorespiratory fitness and autonomic regulation
Van Waart H et al. [40] (2017)	Recruitment to and pilot results of the PACES randomized trial of physical exercise during adjuvant chemotherapy for colon cancer	RCT	23 patients with colon cancer undergoing adjuvant chemotherapy	Low-intensity, home-based program (Onco-Move)-Group 1, Moderate- to high-intensity, combined supervised resistance and aerobic exercise program (OnTrack)- Group 2, Usual Care- Control group.	Feasibility, sociodemographic and clinical characteristics, cardiorespiratory fitness and muscle strength, Fatigue, HRQoL, Physical activity level	Cardiorepiratory fitness, muscle strength and physical activity levels improved in both exercise groups, No clear trend in fatigue levels, HRQoL improved in all the patients over time.
May A M et al. [41] (2016)	Cost-effectiveness analysis of an 18-week exercise program for patients with breast and colon cancer undergoing adjuvant chemotherapy: the randomized PACT study	RCT	204 breast cancer patients and 33 colon cancer patients receiving adjuvant chemotherapy	Aerobic and resistance exercises- Experimental group, Usual care- Control group	Costs, Quality-adjusted life years (QALY) and incremental cost-effectiveness ratio	For colon cancer exercise intervention was cost-effective and improvements were seen in QALY, whereas for breast cancer it was not effective.
Vulpen J K et al. [42] (2016)	Effects of an Exercise Program in Colon Cancer Patients undergoing Chemotherapy	RCT	33 colon cancer patients receiving chemotherapy	Aerobic exercise- Experimental group, Usual care- Control group	Fatigue, QOL, physical fitness, anxiety, depression, body weight, and chemotherapy completion rate	Experimental group less physical and general fatigue and higher physical functioning.
Komatsu H et al. [43] (2016)	A self-directed home yoga program for women with breast cancer during chemotherapy: A feasibility study	Prospective Feasibility study	18 patients with breast cancer receiving adjuvant chemotherapy	Yoga intervention	Feasibility, exercise adherence, fatigue	Retention and adherence was good, improvements in cognitive aspects of fatigue.
Wiskemann J et al. [44] (2016)	Effects of 12-week resistance training during radiotherapy in breast cancer patients	RCT	160 patients with breast cancer receiving radiotherapy	Progressive resistance exercise- Experimental group, Group based relaxation training- Control group	Muscle function, anthropometric measures, CRF	Effective in improving upper and lower limb strength, slight increase in hip circumference in the relaxation group, no significant changes in fatigue
Savage P D et al. [45] (2016)	Fitness during Breast Cancer Treatment and Recovery in an Athlete: A Case Study	Case study	1 patient with breast cancer	Type of exercise not mentioned	VO2 peak	Exercise training helps in reversing the decrements of VO2 peak during breast cancer treatment.
Anne Marie L et al. [46] (2015)	Exercise A Path to Wellness During Adjuvant Chemotherapy for Breast Cancer?	Descriptive and exploratory	27 women who had received exercises during adjuvant chemotherapy	Strengthening exercises with resistance bands and 30 min of brisk walking	Focused group interviews	Shapes feelings of psychological wellness, stimulates feelings of physical wellness, and influences social wellness

Scope and Variety of Articles Published on Cancer Rehabilitation

There was a huge diversity in the literature available. Most of the exercise interventions were given by physiotherapists and exercise physiologists, and even nursing professionals were included. Along with exercises, diet, and nutritional adjustments were also given as a major line of treatment in the intervention groups. This indicated the need for exercise, which, when combined with nutrition, gives better results. Many studies are available on the exercise interventions for cancer survivors after the completion of cancer treatment. But there is less evidence for exercise interventions during ongoing adjuvant therapy. Systematic reviews were also available for the same. But as the target population was specifically cancer patients undergoing adjuvant therapy, those articles were excluded. A total of 27 out of the total 40 articles included were RCTs, two were non-randomized trials, three were prospective interventional studies, one was a survey, three were case studies, one was a case series, one was a pragmatic follow-up study, two were pilot studies, and two were descriptive studies. Out of the articles included, 28 studies were on breast cancer patients, one was on ovarian cancer, 15 were on colon cancer, one was on esophageal cancer, one was on glioblastoma, one was on buccal mucosa cancer, and one was on pancreatic cancer. Out of these, some studies had utilized both colon and breast cancer patients. Most of the studies are from foreign countries, which indicates the need for such studies in India. Most of the trials were funded, and some of the trials are still ongoing, like the DISCO trial, ECHO trial, and many more. These ongoing trials have not been included in this review.

Types of Exercise Interventions

**Figure 2:**
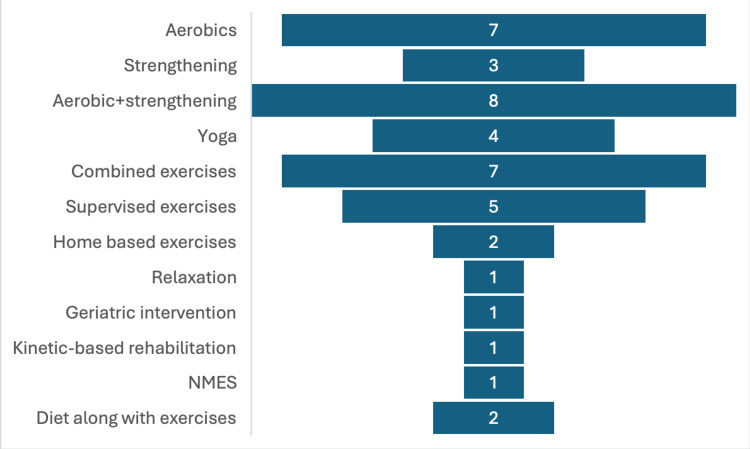
Graphical presentation of the exercise interventions used in the study.

There was a wide variety in the exercise interventions used for cancer patients during ongoing chemotherapy. Circuit interval training in the form of aerobics and strengthening was the most used intervention; combined effects of aerobics and strengthening were also seen. Also, a combination of exercises that included aerobics, strengthening, mobility, balance, and flexibility exercises was used. Yoga therapy is also one of the recent trends in cancer care. Home-based exercises, relaxation, geriatric interventions, kinetic-based rehabilitation, and neuromuscular electrical stimulation (NMES) were less commonly used (Supplementary Table 1). Diet, along with exercise, was included in two studies. The graphical presentation of the same is shown in Figure 2. 

Common Outcome Measures

**Figure 3:**
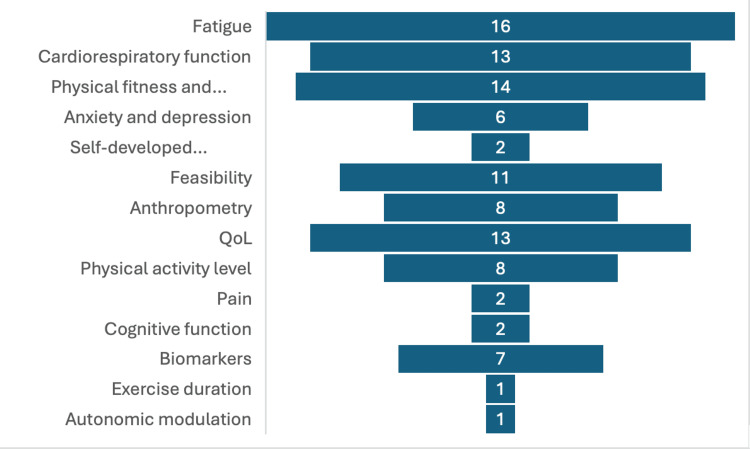
Graphical presentation of the outcome measures used in the study.

Fatigue, cardiorespiratory fitness, and QoL were the frequently used outcomes, followed by physical fitness and function, anthropometric measurements, feasibility, physical activity level, anxiety and depression, biomarkers of inflammation, cognitive function, pain, exercise duration, self-developed questionnaires or interviews, and autonomic modulation. The graphical presentation of the above data is depicted in Figure 3. 

Discussion 

This scoping review provides a comprehensive overview of current trends in exercise interventions for patients with cancer undergoing adjuvant therapy. The primary objective was to map the breadth and diversity of available literature and to identify commonly used exercise interventions and associated outcomes in this population. The findings indicate that randomized controlled trials (RCTs) constitute the majority of the available evidence, followed by prospective interventional studies, case studies, pilot studies, and pragmatic follow-up studies. A considerable proportion of these trials were funded, with some still ongoing, reflecting growing research interest in this field. Most studies were conducted in patients with breast cancer, followed by colorectal and gastroesophageal cancers, whereas cancers such as ovarian, pancreatic, buccal mucosa, and glioblastoma were underrepresented.

Cancer-related fatigue emerged as one of the most frequently reported symptoms, arising either from the disease itself or as a side effect of treatment [47]. Additionally, anticancer therapies were associated with reduced cardiorespiratory function and overall fitness, potentially increasing the risk of cardiovascular complications [48]. Psychological concerns, particularly anxiety and depression, were also highly prevalent among patients [49]. Collectively, these factors contribute to a significant decline in quality of life (QoL) [3]. Accordingly, the most commonly assessed outcomes across studies included fatigue, QoL, and cardiorespiratory fitness, followed by physical activity levels, anthropometric measures, psychological outcomes, and feasibility.

Exercise interventions demonstrated substantial benefits in improving physical function and overall fitness, including muscle strength, endurance, and cardiovascular capacity. These improvements are partly attributed to enhanced regulation of energy metabolism, which contributes to better physical performance [50]. Improved physical capacity can also foster greater confidence and a sense of well-being, thereby alleviating anxiety and depression and enhancing QoL. The most commonly implemented interventions included aerobic exercises, resistance training, and combined exercise programs. Several studies also employed personalized, multimodal protocols incorporating aerobic, resistance, balance, mobility, and flexibility training, highlighting the importance of individualized exercise prescription.

In addition to conventional exercise approaches, yoga has emerged as a promising complementary therapy. It is believed to regulate the autonomic nervous system by balancing sympathetic and parasympathetic activity, thereby helping to alleviate cancer-related symptoms [51]. This has contributed to its increasing incorporation into cancer care programs.

Despite these positive findings, challenges related to feasibility were frequently reported. Issues such as poor adherence and loss to follow-up were common across studies. To address these barriers, patient education regarding the benefits of exercise should be emphasized. Providing structured training prior to intervention initiation and incorporating engaging and interactive exercise formats, as demonstrated in a case report by Nair T et al. [9], may further enhance adherence. These considerations are particularly important when designing large-scale clinical trials.

Several limitations were identified within the included studies, including small sample sizes, heterogeneity in participant characteristics, and insufficient reporting of exercise intensity and duration. Additional challenges included time constraints and patient convenience, which may have influenced participation and outcomes. Addressing these limitations in future research will be essential for improving the quality and applicability of evidence.

This scoping review itself has certain limitations. It adopted a broad approach by including all types of exercise interventions and outcomes, but excluded cancer survivors and patients undergoing neoadjuvant therapy. The literature search was limited to two databases, and no critical appraisal of included studies was conducted, consistent with standard scoping review methodology [7]. Furthermore, there was a paucity of evidence from India, indicating a need for region-specific research. Future studies should consider conducting detailed systematic reviews with more focused research questions and expanded database searches to strengthen the evidence base.

Importantly, to enhance clinical applicability, future work should aim to translate these findings into a concise, practical framework that provides clear, actionable exercise recommendations for clinicians managing patients during adjuvant therapy. Such efforts would support the integration of exercise into routine oncology care and promote patient-centered rehabilitation.

Clinical implications

Exercise interventions can be safely integrated into the care of patients undergoing chemotherapy or radiotherapy to help manage treatment-related symptoms such as fatigue, reduced physical fitness, anxiety, and depression, thereby enhancing overall quality of life. To support clinical implementation, these interventions can be translated into a concise, practical framework outlining clear, actionable recommendations for clinicians managing patients during adjuvant therapy. Such a framework may include simple, individualized aerobic and resistance exercise prescriptions, such as home-based activities like walking, tailored to patient capacity, thereby facilitating multidisciplinary cancer rehabilitation and promoting patient self-management during treatment.

## Conclusions

This scoping review gives a detailed overview of the different types of exercise interventions and outcomes used in patients with cancer undergoing adjuvant therapy. Aerobic and strengthening exercises were the most used, followed by combination exercises and yoga therapy. The frequently used outcomes included fatigue, cardiorespiratory fitness, and QoL, followed by physical fitness, anxiety, and depression. Very few studies were from India, and looking at the prevalence of cancer in India, there is a large scope for cancer research in India.
